# Post-Operational Photodynamic Therapy of the Tumor Bed: Comparative Analysis for Cold Knife and Laser Scalpel Resection

**DOI:** 10.3390/biomedicines12020291

**Published:** 2024-01-26

**Authors:** Maria Shakhova, Vadim Elagin, Anton Plekhanov, Aleksandr Khilov, Daria Kurakina, Vladislav Kamensky, Mikhail Kirillin

**Affiliations:** 1 Department of Ear, Nose and Throat Diseases, FSBEI HE «Privolzhsky Research Medical University» MOH Russia, 10/1 Minin and Pozharsky Square, Nizhny Novgorod 603005, Russia; maha-shakh@yandex.ru; 2 Institute of Experimental Oncology and Biomedical Technologies, FSBEI HE «Privolzhsky Research Medical University» MOH Russia, 10/1 Minin and Pozharsky Square, Nizhny Novgorod 603005, Russia; strike_gor@mail.ru (A.P.); vlad@ufp.appl.sci-nnov.ru (V.K.); 3 A.V. Gaponov-Grekhov Institute of Applied Physics of the Russian Academy of Sciences, 46 Ulyanov St., Nizhny Novgorod 603155, Russia; alhil@inbox.ru (A.K.); vekfy@inbox.ru (D.K.); mkirillin@yandex.ru (M.K.)

**Keywords:** photodynamic therapy, tumor resection, laser surgery, cold knife, chlorin-based photosensitizers, Monte Carlo simulations

## Abstract

In this paper, we report on a study regarding the efficiency of the post-operational phototherapy of the tumor bed after resection with both a cold knife and a laser scalpel in laboratory mice with CT-26 tumors. Post-operational processing included photodynamic therapy (PDT) with a topically applied chlorin-based photosensitizer (PS), performed at wavelengths of 405 or 660 nm, with a total dose of 150 J/cm^2^. The selected design of the tumor model yielded zero recurrence in the laser scalpel group and 92% recurrence in the cold knife group without post-processing, confirming the efficiency of the laser scalpel in oncology against the cold knife. The application of PDT after the cold knife resection decreased the recurrence rate to 70% and 42% for the 405 nm and 660 nm procedures, respectively. On the other hand, the application of PDT after the laser scalpel resection induced recurrence rates of 18% and 30%, respectively, for the considered PDT performance wavelengths. The control of the penetration of PS into the tumor bed by fluorescence confocal microscopy indicated the deeper penetration of PS in the case of the cold knife, which presumably provided deeper PDT action, while the low-dose light exposure of deeper tissues without PS, presumably, stimulated tumor recurrence, which was also confirmed by the differences in the recurrence rate in the 405 and 660 nm groups. Irradiation-only light exposures, in all cases, demonstrated higher recurrence rates compared to the corresponding PDT cases. Thus, the PDT processing of the tumor bed after resection could only be recommended for the cold knife treatment and not for the laser scalpel resection, where it could induce tumor recurrence.

## 1. Introduction

The introduction of laser scalpels into oncological surgery in recent decades [1,2] was a significant advance, ensuring R0 resection, owing to their efficient cutting capabilities combined with tissue carbonization, a considerable reduction in bleeding, minimized intraoperative trauma, and the absence of coarse scars in the postoperative period. Diode lasers have been shown to reduce tumor dissemination during surgical procedures due to bleeding prevention [3,4]. However, postoperative local recurrences still remain an important problem in the clinical treatment of cancers. In this connection, the additional improvement in surgical protocols to additionally decrease the risk of further tumor growth, and the probability of recurrence is of high importance [5]. In particular, a protocol including a procedure allowing for the elimination of cancer cells left in tissue after surgical intervention could be advantageous against traditional approaches. A combined approach to the treatment of tumors of different localizations may include photodynamic therapy (PDT) [6,7,8], antitumor immunity [9,10], cryotherapy [11,12,13], hyperthermia [14,15], and radiotherapy [16,17,18], in addition to surgical treatment, which helps to reduce the recurrence rate, and this can not only increase the survival period but also improve the quality of life of cancer patients [19]. In addition, considering the possibility of invasive tumor growth and the spread of tumor cells beyond the visually defined resection margin, as well as the fact that surgical intervention itself does not provide ablasticity, improvements in both the surgical protocols and the introduction of additional intraoperative treatment methods would ensure R0 resection is relevant.

Photodynamic therapy is a low-invasive modern treatment technique based on the photoactivation of a photosensitizer, resulting in the production of singlet oxygen and other reactive oxygen species, and which has proved to be efficient in oncology [20,21]. This technique does not require accurately targeting cancer cells and provides action to the areas with an accumulated photosensitizer (PS) irradiated with light of a specific wavelength. Thus, the configuration of the irradiation beam determines the shape of the treated volume. The PDT intraoperatively used for the treatment of the tumor bed to control the remaining cells of the primary tumor and reduce the risk of recurrence was first described in the 1990s [22]. When performing PDT in oncology, the intravenous administration of a photosensitizer is traditionally used. It should be noted that even a minimal accumulation of PS in the skin can trigger a photochemical reaction under the influence of daylight and lead to the development of complications after the PDT procedure in the absence of preventive measures. These limitations, as well as the economic and time aspects of the PDT procedure, have led to the development of topical formulations of PS with more favorable photophysical properties in the form of ointments, solutions, and gels.

Topically applied PSs do not require intravenous injection, and they benefit from a simple application procedure [23]. Chlorin-based photosensitizers feature two peaks in their absorption spectrum in the red and blue bands, thus providing different penetration depths owing to significant differences in the biotissue optical properties of these bands [24,25]. Previously, PDT with topically applied PS has demonstrated limited efficiency in tumor treatment, owing to the limited penetration of PS into the tumor [26]; however, its application to the tumor bed after the procedure seems to be promising. The choice of blue or red light for irradiation provides the opportunity for performing strong superficial action or a deeper action with weaker depth dependence based on the evaluation of the tumor cell invasion depth. Numerical Monte Carlo simulations have demonstrated significant differences in the in-depth distribution of the absorbed light dose governing PDT treatment areas for blue and red bands [25].

The introduction of PDT gels [27] has raised the question regarding the additional treatment of the surgical tumor bed during organ-preserving operations [28,29]. In this study, we compared the result of using PDT after the local application of the PS gel after tumor excision using a cold knife and a contact laser scalpel with an optical fiber that has the shape of a knife. The theory of “correct geometry” and the development of the methodology of the contact laser scalpel application have recently been developed [30]. An important feature is the contact laser cutting, which seals blood vessels and results in the absence of bleeding, as well as the minimization of the tumor cell spread into healthy tissue when working in ablative mode. The main criterion for treatment efficiency, including the surgical removal of the tumor, is the absence of continued growth and recurrences over long periods, with an assessment of the five-year survival rate. Different coagulation properties of the wound surface after contact laser surgery and cold knife tumor resection, in particular, the different diffusions of the PDT gel into the biological tissue, stimulate the comparison of a combination of these techniques with PDT for the post-operative elimination of the remaining cancer cells.

The aim of this study was to compare the effect of additional PDT of the tumor bed with topical PS application after using a cold knife (no coagulation layer) and after contact excision with a waveguide laser scalpel operating at 0.97 μm, forming a coagulation layer with a thickness of about 300 μm [31]. This study was performed with a chlorin-based PS in order to additionally compare the effects of the PDT procedure at 405 and 660 nm. The invasion of the employed tumor model into the muscular tissues beyond the tumor node was verified by histology. The distribution of the PS in tissue upon administration was analyzed using fluorescence microscopy, while the distribution of the absorbed light dose at the considered wavelengths was studied using Monte Carlo simulations.

## 2. Materials and Methods

### 2.1. Animal Cohort

All animal studies were approved by the Ethics Committee of the Privolzhsky Research Medical University (Protocol No. 13 of 7 July 2021). Experiments were performed on 113 female Balb/c mice. Mice were subcutaneously inoculated in the left flank with mouse colon adenocarcinoma (CT-26) cells (in an amount of 2.5 × 10^5^ cells per injection) in 100 µL of phosphate-buffered saline. All animals were blindly divided into 10 experimental groups according to the type of post-operational treatment of the tumor bed (Table 1). The perioperative death cases during the experiment in different groups occurred within 2 h after the procedure and were caused by the complications of the narcotization procedure.

### 2.2. Resection Procedure

Tumors were resected on the 10th day after inoculation, when the tumor node volume had reached approximately 1 cm^3^. The mice were anesthetized intramuscularly with a mixture of Zoletil (40 mg/kg, Virbac, Carros, France) and 2% Rometar (10 mg/kg, Spofa, Jičín, Czech Republic) before the tumor node resection. The skin on the tumor site was cut using a cold knife. The tumor nodes were resected using either a commonly used cold knife or a laser scalpel. An LSP-0.97/10 (IRE-Polyus, Fryazino, Russia) laser scalpel operating at a wavelength of 0.97 µm with an output power of 6 W was used to excise the tumor. The laser light was delivered to the tissue via a silica fiber of 550 µm in diameter. The surgeon removed the tumor node in accordance with visual evaluation of tumor margins.

### 2.3. PDT Procedure

To perform a photodynamic therapy procedure, a chlorin e6-containing gel (Radagel^®^, ”RADA-PHARMA” Ltd., Moscow, Russia) was used. The gel contains the sodium salt of chlorin e6 as an active substance in concentration of 0.0175 mg per 10 mL. Approximately 100 mg of the gel was topically applied onto the surface of the tumor bed by a cotton swab and was evenly distributed over the surgical field. The gel was kept for 20 min and then removed with wet gauze pads. In the course of the PDT procedure of the tumor bed after the tumor excision, the irradiation was performed with the PDT device “Harmonia” (Laser MedCenter Ltd., Moscow, Russia) with a total dose of 150 J/cm^2^; the fluence rate at the tissue surface was 260 and 125 mW/cm^2^ for the wavelengths of 660 and 405 nm, respectively. The irradiation spot size was 9 mm in diameter. The fluence rate was different for 660 and 405 nm since our previous studies indicated that the equal fluence rate results in extensive heating of the tumor site in course of irradiation at a wavelength of 405 nm. On the other hand, we wanted to minimize the procedure time and limit the irradiation procedure to within half an hour to prevent mice from waking up from narcosis during irradiation. The irradiation-only treatment was performed in the same conditions, with the same device and doses, as the PDT treatment, though without application of the PS gel.

### 2.4. Follow-Up

The appearance of tumor recurrence was monitored for 3 months after the resection. Upon completion of the monitoring or detection of tumor recurrence reaching 1 cm^3^ in size, the animals were sacrificed. All animal procedures followed the Guidelines for Works Involving Experimental Animals and the International Guiding Principles for Biomedical Research Involving Animals and the ethical principles established by the European Convention for the Protection of Vertebrate Animals used for Experimental and other Scientific Purposes.

For comparative analysis, we tried to standardize the experiment; the resection was carried out by one surgeon, the mice were from the same series, and the PDT procedure was carried out with the same device and personnel.

### 2.5. Analysis of Photosensitizer Distribution

A study was performed in order to analyze the penetration of the PS into the tumor bed upon topical application. The skin above the tumor node and the part of the tumor of about 3 mm in thickness were removed using a cold knife or a laser scalpel. The study involved 5 animals for each resection technique. The Radagel PS was applied onto the obtained surface and the animals were kept in a dark box. After 20 min, the PS was removed from the surface and the tumors were excised. The excised tumors were embedded in O.C.T. compound and immersed in ethanol cooled to −80 °C. Twenty-micrometer-thick cryosections oriented normal to the tissue surface were prepared using a bench-top cryostat CM 1100 (Leica Biosystems, Nussloch, Germany) at a temperature of −20 °C. To avoid redistribution of the PS on the slice surface, the tumor samples were oriented in such a way that the cryostat knife reached the surface of the Radagel administration after passing through the entire sample. Fluorescence intensity of cryosections was studied using laser-scanning microscope LSM 880 (Carl Zeiss, Jena, Germany). An oil-immersion objective C-Apochromat 40×/1.45 NA was used for image acquisition. Chlorin e6 fluorescence was excited at a wavelength of 633 nm with a He-Ne laser. Fluorescence emission was detected in the band of 650–735 nm. For each sample, 12 tile scans from 4 cryosections were acquired. The tile scan consists of 6 serial images captured consequently, starting from the sample surface. The fluorescence intensity images were processed with ZEN 3.0 (Carl Zeiss) and ImageJ 1.39p software (NIH, Bethesda, MD, USA).

### 2.6. Monte Carlo Simulations

In order to interpret the results of the experiment, we performed a simulation of the distribution of the absorbed dose within tissue at two employed wavelengths. Simulations of the absorbed light distribution were conducted with the use of our previously reported code [32] for Monte Carlo modeling of light transport in biological tissues. To analyze the absorbed light distribution, models of tumor and muscle mouse tissues were considered. The simulations were performed for uniform illumination of the tissue sample at wavelengths of 405 and 660 nm. It is worth noting that the optical properties data reported for mouse tumor models as well as mouse muscle tissues are quite limited. Absorption and reduced scattering coefficients for tumor tissue model were chosen in accordance with [33,34]. Due to the lack of literature data, optical properties of mouse muscle tissue were reconstructed from our ex vivo spectrophotometric measurements [35] using the look-up-table method described in [36]. The considered optical properties for the wavelengths of 405 and 660 nm are summarized in Table 2.

For the considered optical properties of the tissue model, a 3D map of the absorbed light dose $A\left(x,y,z,\lambda \right)$ with a wavelength *λ* was calculated and transformed into the in-depth density of the absorbed dose fraction:

$$\tilde{A\;}\left(z,\lambda \right)=\iint \frac{A\left(x,y,z,\lambda \right)}{dVN_{ph}}dx\;dy\;$$

normalized to the value of $N_{ph}dV$ [32], where $N_{ph}=10^{7}$ is the total number of launched photons, and dV is the volume of a voxel.

## 3. Results and Discussion

### 3.1. Histology Study

**Figure 1:**
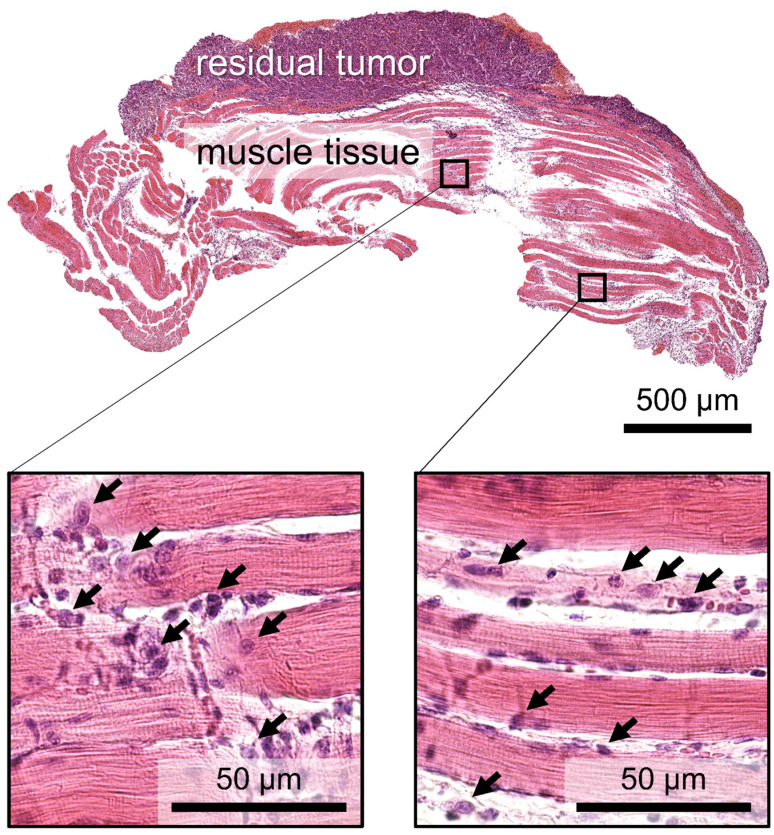
Histological image of the tumor bed after removal with a cold knife. The arrows show revealed tumor cells.

Since the aim of this study was to compare the efficiency of different treatment protocols in suppressing tumor growth and preventing recurrence, it was important to choose the model with the high probability of tumor invasion into muscular tissue, when accurate surgical removal of tumor node with a cold knife may not ensure R0 resection [37]. In order to evaluate the adequacy of the chosen model, we performed a histological study of a typical tumor on the 10th day after inoculation. An image of a hematoxylin–eosin (H&E)-stained tissue sample showing the tumor node and underlying muscular tissue is shown in Figure 1. This figure demonstrates the invasion of tumor cells into the layer of muscular tissue beneath the tumor node. Two arbitrary areas within the muscular layer shown with higher magnification clearly demonstrate tumor cells located between muscle fiber bundles as well as disorganization of the bundle structure owing to tumor proliferation. The observed approval of the high tumor cell count outside the tumor node indicates the adequacy of the chosen model with respect to the aims of the study. Note that the tumor cells could be found as deep as 1 mm below the tumor node margin.

### 3.2. Fluorescence Microscopy

**Figure 2:**
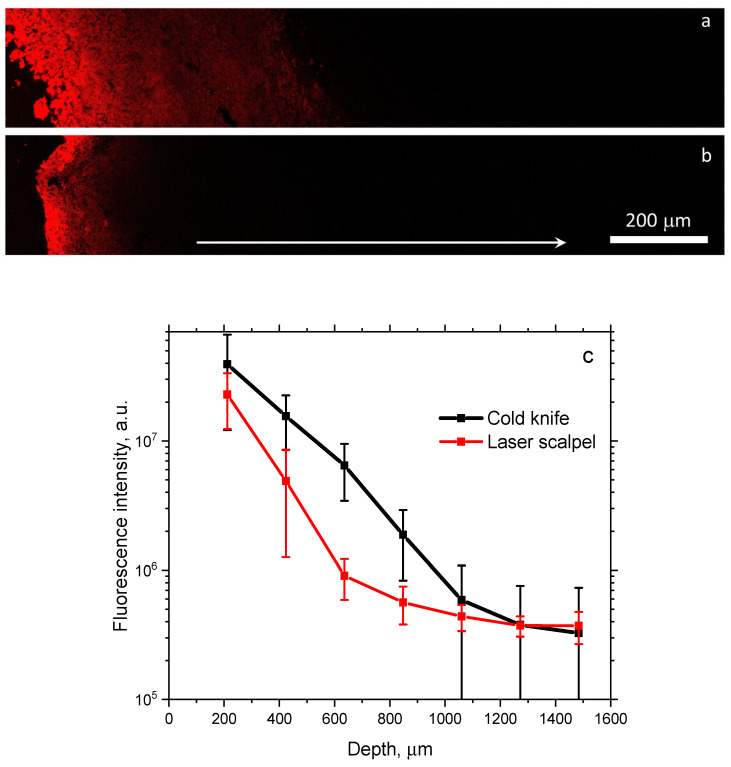
Fluorescence microscopy images of the in-depth PS distribution upon topical application on a tumor after surgical removal of superficial tumor layer with the cold knife (**a**) and the laser scalpel (**b**). White arrow shows in-depth direction. Depth dependence of PS fluorescence upon topical application in muscular tissue within the tumor bed after tumor resection with the cold knife and the laser scalpel (**c**). Graphs show mean values ± standard deviation for 5 animals in each group.

In our previous study, we demonstrated a high efficiency of employing a laser scalpel instead of a cold knife in preventing tumor recurrence [38], while the aim of this study was to evaluate the additional effect of a complementary PDT procedure on the tumor bed after surgical treatment. As demonstrated earlier, the use of a laser scalpel results in the formation of a caramelization layer [39] on the tissue surface, the parameters of which are described in other papers [30,31]. The studies [30,31] demonstrated that the thickness of this layer is usually about 300–400 µm. One may expect the difference in the penetration of a topically applied PS into tissue after resection with the cold knife and the laser scalpel owing to the differences in the properties of this layer. In order to demonstrate this difference, an analysis of the PS distribution upon topical application was performed using fluorescence microscopy images of tumor cross-sections stitched from several fields of view. Figure 2 shows typical fluorescence microscopy images, corresponding to the cold knife (Figure 2a) and laser scalpel (Figure 2b) tumor resection. One can see that the PS penetrates much deeper into tissue in the case of the cold knife resection. The cold knife case demonstrates a pronounced fluorescence signal to depths of about 600 µm, while for the laser scalpel case, this depth is below 200 µm.

In order to quantify the difference in in-depth PS distribution for the two considered surgical options, we plotted the dependencies of the integral fluorescence signal in an image tile on depth averaged over five independent images each. The results of the fluorescent PS in-depth distribution analysis are shown in Figure 2c on the logarithmic scale. The observed dependencies confirm the exponential in-depth decay of the PS concentration for both cases, which is in agreement with the predictions of the analytical model of substance diffusion into tissue [40]. Comparisons of the two dependencies indicate both a lower fluorescence signal from the laser scalpel-cut tissue and faster in-depth attenuation of the PS concentration. The thickness of the caramelization layer was estimated previously, amounting to about 400 µm, so the dependence shows that the fluorescence signal from the depth of 636 µm can be assumed to correspond to a depth below the caramelization layers that is more than 20-times smaller compared to the signal from the superficial layer. At the same time, the corresponding attenuation of the fluorescence signal for the cold knife is less than 7-times. The observed dependencies indicate the difference in the in-depth PS concentration distribution between two considered cases, so one can expect deeper PDT action for the cold knife option. On the other hand, it is worth noting that the concentration reaches an asymptotic nature at a depth of about 1 mm, allowing us to assume that, at least in some cases, the concentration of PS at this depth is negligible. This fact allows one to expect that the PDT action at this depth could be insufficient to suppress tumor cells. Several studies have previously demonstrated that low-dose PDT [41] or topical administration of PS [26] may result in an incomplete response of a tumor to treatment. Moreover, it was demonstrated earlier that low-dose PDT may provoke tumor recurrence [42] or increase in metastatic activity [43].

### 3.3. Monte Carlo Simulations 

**Figure 3:**
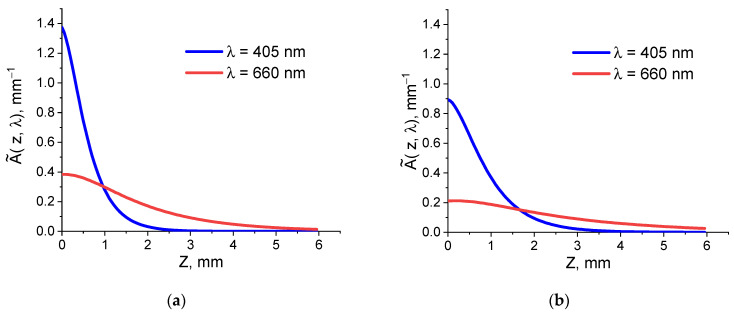
In-depth profiles of the absorbed light dose density for the irradiation wavelengths of 405 and 660 nm for the tumor tissue (**a**) and muscle tissue (**b**) models.

Another factor that may affect the performance of PDT in deeper tissue layers is the penetration depth of irradiation, which is used to activate the accumulated PS. In this study, with the chlorin-based PS, we compare the effect of PDT performance at the wavelengths of 660 and 405 nm, both of which correspond to chlorin e6 absorption peaks [24] and were proposed for PDT performance [26]. Since the absorbed light dose distribution within tissue cannot be measured directly, we performed Monte Carlo simulations of the absorbed dose distribution for the values of optical properties typical for tumors and muscular tissues summarized in Table 2. Figure 3 shows in-depth profiles of the absorbed dose density calculated for the values typical for tumor (Figure 3a) and muscular (Figure 3b) tissue. The profiles for tumor tissue show faster in-depth attenuation, as compared to muscular tissue owing to the average higher blood content in tumors. The density of the absorbed dose is higher for a wavelength of 405 nm to a depth of about 1 mm for tumors and about 1.6 mm for muscles. Despite strong attenuation at a wavelength of 405 nm, the absorbed doses cannot be neglected until depths of above 1.5 mm and much deeper for a wavelength of 660 nm, indicating that the light of both wavelengths penetrates deeper than PS and can potentially affect deep-seated tumor cells located below.

These preliminary studies allow us to conclude that, although PS penetrates quite deep into tissue upon topical application, the caramelization of the superficial tissue layer as a result of the laser scalpel use may prevent deeper PS penetration, thus limiting the impact depth of the PDT procedure. On the other hand, the light at both wavelengths penetrates deeper for both considered models of tumors and muscles. Although these models do not account for the caramelization layer, we do not expect it to significantly affect the optical properties, so these estimates could be treated as reliable for a qualitative analysis.

### 3.4. Animal Study 

**Figure 4:**
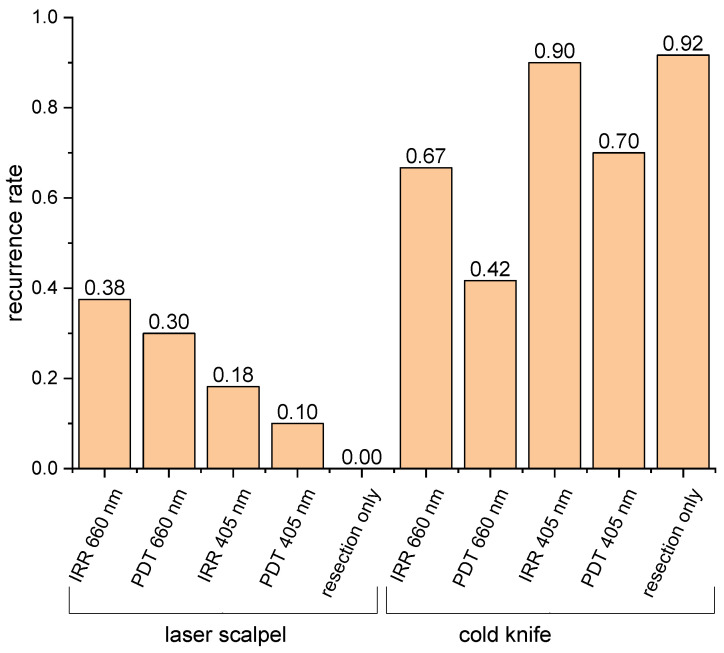
Tumor recurrence rate after surgical removal with the laser scalpel and the cold knife and different regimes of the post-operational PDT of tumor bed (PDT—PDT procedure after topical application of Radagel, IRR—irradiation-only procedure without PS administration).

Typical photographs of laboratory animals prior to the treatment and in the follow-up period for different treatment protocols are shown in the Supplementary Materials (Figure S1). The results of the treatment outcomes in the considered 10 groups of experimental animals (see Table 1) are summarized in Figure 4. The selected design of the tumor model yielded a zero recurrence rate in the laser scalpel group and 92% recurrence in the cold knife group without post-processing, confirming the efficiency of the laser scalpel in oncology against the cold knife. This result also confirms tumor cell invasion into underlying muscular tissues left after the resection procedure. The application of PDT after the cold knife resection decreased the recurrence rate to 70% and 42% for 405 nm and 660 nm PDT procedures, respectively. This fact allows one to assume that either the accumulated concentration of PS was insufficient to provide the desired effect or the penetration depth of PS was inadequate for a full response. The fact of much more efficient suppression of recurrence with PDT at 660 nm indicates that light penetration may play a major role in providing the effects of a PDT procedure, so a deeper PDT impact seems to be the key for a successive prevention of recurrence. The observed effect of the recurrence rate decrease is in line with the results with the same tumor line reported in [6], which demonstrated a recurrence rate of 17–33% after PDT of the tumor bed against 83–100% recurrence without PDT. It is worth noting that the study of [6] was performed with another type of PS administered intravenously, which supposedly resulted in a better prevention of recurrence, as compared to our study. Moreover, in that study, wavelengths of 630 and 510 nm were employed for the PDT procedure, providing deeper action, as compared to a wavelength of 405 nm, which demonstrated a weak suppression of recurrence in our study.

Surprisingly, the application of PDT after the laser scalpel resection induced recurrence rates of 18% and 30%, respectively, for wavelengths of 405 and 660 nm at the background of total absence of recurrences in the laser-scalpel-only group. This observation allows for assuming that, owing to the limited penetration of PS governed by the presence of the caramelization layer with comparable light penetration, the stimulation of deeper tumor cells may occur. This assumption is confirmed by the observations in the irradiation-only control groups, when post-operation treatment consisted of light irradiation without PS application. In all four irradiation-only groups, the recurrence rate is higher than that in the corresponding PDT groups, indirectly confirming the assumption regarding the stimulation of tumor growth by light in the absence of PS.

However, one can see that the recurrence rate in the irradiation-only group at 405 nm is comparable with the control group, while for the irradiation-only group at 660 nm, it is considerably smaller, which allows us to conclude on the competing processes of suppression and stimulation of tumor recurrence. Supposedly, laser light may suppress recurrence in upper tissue layers, where the fluence rate is rather high, while the low fluence rate may stimulate further tumor development at depth. The potential of the laser light in the partial suppression of tumor growth is also indirectly confirmed by our earlier studies [26], which indicated a delay in tumor growth after irradiation at the employed wavelengths.

On the other hand, previous studies showed that a low-level light illumination may stimulate the proliferation of osteosarcoma, lung carcinoma cells [44], and oral carcinoma cells [45]. This treatment may also modulate inflammation by attenuating TNF-α/cycloheximide-induced apoptosis with a reduction in caspase-3/7/8/9 [46]. It is also worth noting that for the laser scalpel groups, where PS penetration is weaker, the wavelength of 660 nm characterized by deeper penetration provides a higher recurrence rate, as compared to the wavelength of 405 nm, for which the penetration is limited. The cold knife groups demonstrate the opposite effect at the background of deeper PS penetration, where insufficient penetration of irradiation at a wavelength of 405 nm seems to be more critical in providing recurrence rates for the irradiation-only group at 405 nm, comparable with that for the cold knife control group. One can also suppose that the formation of the caramelization layer eliminated the remaining tumor cells in this layer, and recurrence may occur in the tissue below this layer. In this connection, red light, which penetrates deeper, has a higher potential for recurrence stimulation, as compared to blue light at the background of light attenuation in the caramelization layer that results in a low-dose light impact of the underlying tissue.

## 4. Conclusions

In this paper, we analyzed the efficiency of PDT of the tumor bed as a tool to prevent tumor recurrence after tumor surgical removal. We compared both cold knife and laser scalpel resection, while the choice of the topically applied chlorin-based photosensitizer allowed us to study PDT with red or blue light, which differ significantly in penetration depth in tissue. The initial idea for this study was to provide an additional impact to tumor cells that could remain in tissues after tumor removal. Preliminary fluorescence microscopy studies indicated a significant difference in the distribution of PS in tissues upon topical application for the cold knife and laser scalpel surgery, supposedly owing to the formation of a caramelization layer after the laser surgery. This difference is manifested by a much deeper penetration of PS after the cold knife surgery. Preliminary analysis of the light penetration depth at the two considered wavelengths performed using numerical Monte Carlo simulations indicated deeper penetration of light, as compared to PS penetration in tissues, which allowed us to assume that light could impact deep-seated tumor cells that were not reached by PS. The results of the experiment confirmed some assumptions regarding the effect of PDT of the tumor bed on the tumor recurrence rate; however, some of the revealed effects are surprising. As expected, PDT provided a decrease in the tumor recurrence rate after the cold knife surgery, and the red-light PDT provided a better effect, as compared to the blue-light PDT, owing to the deeper penetration of red light. The irradiation-only groups demonstrated a higher recurrence rate for both red and blue light, additionally confirming the positive effect of PDT.

However, PDT after laser scalpel treatment demonstrated the opposite effect; in contrast to the absence of recurrences in the laser-surgery-only group, both the red- and blue-light PDT after laser scalpel resection demonstrated a certain level of recurrence. This is presumably associated with light stimulation of the remaining tumor cells in the background of weaker penetration of PS through the caramelization layer upon topical application. Higher recurrence rates in the irradiation-only groups after the laser scalpel resection as compared to the corresponding PDT groups confirm the stimulating impact of irradiation in the absence of PS. Thus, one should be careful when deciding to improve the surgical tumor removal with post-operational PDT. It seems that a sufficient delivery of PS to the remaining tumor cells should be the key element of this procedure. Perhaps some additional techniques should be employed to confirm sufficient accumulation of PS in tissue depth; alternatively, a traditional approach of intravenous PS injection should be considered.

## Figures and Tables

**Table 1:** Groups of experimental animals depending on the type of resection and post-operative treatment of the tumor bed (numbers in parentheses show perioperative deaths).

Group No.	Resection Method	Treatment of the Tumor Bed	Number of Animals
1	laser scalpel	Irradiation only @660 nm	11 (3)
2	laser scalpel	PDT: Radagel^®^ @660 nm	11 (1)
3	laser scalpel	Irradiation only @405 nm	11 (-)
4	laser scalpel	PDT: Radagel^®^ @405 nm	11 (1)
5	laser scalpel	No additional treatment	11 (-)
6	cold knife	Irradiation only @660 nm	11 (2)
7	cold knife	PDT: Radagel^®^ @660 nm	11 (-)
8	cold knife	Irradiation only @405 nm	10 (-)
9	cold knife	PDT: Radagel^®^ @405 nm	11 (1)
10	cold knife	No additional treatment	13 (1)
	Total		113 (9)

**Table 2:** Absorption $\mu_{a}$ and reduced scattering $\mu_{s}’$ coefficients and reflective index *n* used in Monte Carlo simulations.

Wavelength, nm	$\mu_{a},\;{mm}^{-1}$	$\mu_{s}’,\;{mm}^{-1}$	*n*
	Tumor tissue		
405 nm	0.92	1.29	1.4
660 nm	0.18	0.67	1.4
	Muscle tissue		
405 nm	0.57	1.17	1.4
660 nm	0.07	0.58	1.4

## Data Availability

The data presented in this study are available on request from the corresponding author.

## Supplementary Materials

The following supporting information can be downloaded at: www.mdpi.com/xxx/s1, Figure S1: Photographs of laboratory animals before tumor resection (upper row) and in the follow up (lower row). Animals with recurrences (IRR 660 nm for laser scalpel and all examples for cold knife) were documented 3 weeks after resection. Animals without recurrences were documented 3 months after resection (examples for laser scalpel, except IRR 660 nm). The treatment protocols are indicated under each column (IRR = irradiation only, PDT = PDT procedure, irradiation wavelength is shown next to the treatment option, the irradiation dose was 150 J/cm^2^).
