# Supplementary material for: Post-Operational Photodynamic Therapy of the Tumor Bed: Comparative Analysis for Cold Knife and Laser Scalpel Resection
Source: Biomedicines. 2024 Jan 26;12(2):0. doi: 10.3390/biomedicines12020291 (PMC11154242; doi:10.3390/biomedicines12020291)
Supplement: Supplementary file 1 [file biomedicines-12-00291-s001.zip › biomedicines-2823102-supplementary.pdf]

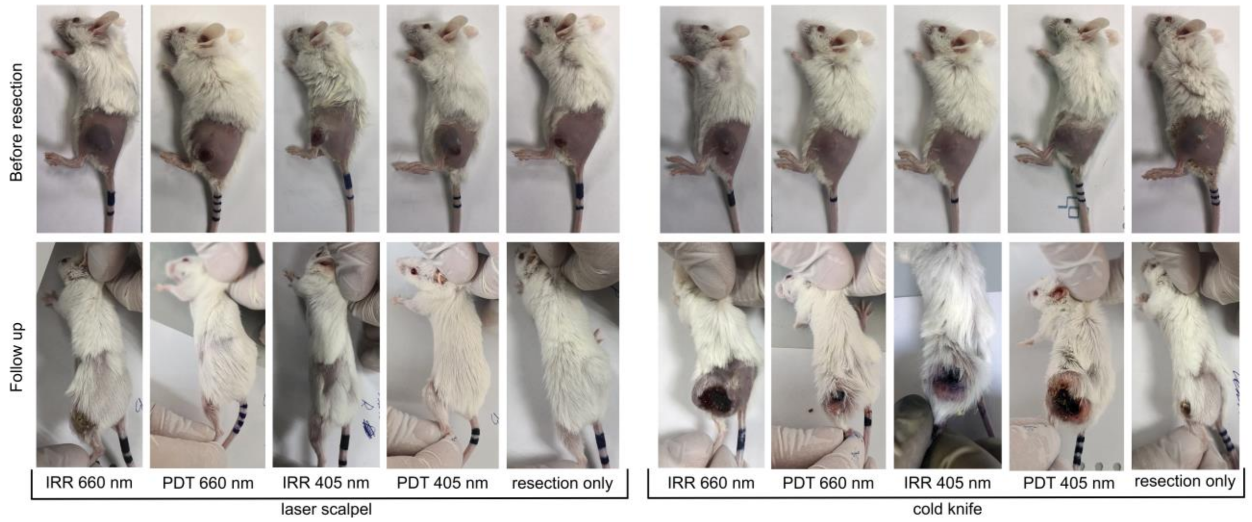


**Figure 1S.** Photographs of laboratory animals before tumor resection (upper row) and in the follow up (lower row). Animals with recurrences (IRR 660 nm for laser scalpel and all examples for cold knife) were documented 3 weeks after resection. Animals without recurrences were documented 3 months after resection (examples for laser scalpel, except IRR 660 nm). The treatment protocols are indicated under each column (IRR = irradiation only, PDT = PDT procedure, irradiation wavelength is shown next to the treatment option, the irradiation dose was 150 J/cm$^2$).
